# A Potential Role for Bat Tail Membranes in Flight Control

**DOI:** 10.1371/journal.pone.0018214

**Published:** 2011-03-30

**Authors:** James D. Gardiner, Grigorios Dimitriadis, Jonathan R. Codd, Robert L. Nudds

**Affiliations:** 1 Faculty of Life Sciences, University of Manchester, Manchester, United Kingom; 2 Département d'aérospatiale et mécanique, Université de Liège, Liège, Belgium; University of Western Ontario, Canada

## Abstract

Wind tunnel tests conducted on a model based on the long-eared bat *Plecotus auritus* indicated that the positioning of the tail membrane (uropatagium) can significantly influence flight control. Adjusting tail position by increasing the angle of the legs ventrally relative to the body has a two-fold effect; increasing leg-induced wing camber (i.e., locally increased camber of the inner wing surface) and increasing the angle of attack of the tail membrane. We also used our model to examine the effects of flying with and without a tail membrane. For the bat model with a tail membrane increasing leg angle increased the lift, drag and pitching moment (nose-down) produced. However, removing the tail membrane significantly reduced the change in pitching moment with increasing leg angle, but it had no significant effect on the level of lift produced. The drag on the model also significantly increased with the removal of the tail membrane. The tail membrane, therefore, is potentially important for controlling the level of pitching moment produced by bats and an aid to flight control, specifically improving agility and manoeuvrability. Although the tail of bats is different from that of birds, in that it is only divided from the wings by the legs, it nonetheless, may, in addition to its prey capturing function, fulfil a similar role in aiding flight control.

## Introduction

In recent years it has become established that bird tails have important effects upon their flight. For example, bird tails are known to produce lift during flight [1], [2]. Bird tails also appear to reduce body drag, by acting as a splitter plate [3] that reduces flow separation behind the body, essentially making the body more streamlined [4]. Furthermore, sufficient flight stability is essential for all flying animals and bird tails are thought to be a key component for overall flight stability [5], [6], [7]. Bird tails are also thought to be important for flight control, particularly during take-off and landing when the tail is fanned out and the angle of attack increased, augmenting lift production, improving manoeuvrability and possibly reducing wing stall [8], [9].

In contrast to birds, relatively little research has investigated the aerodynamic function(s) of a bat's tail membrane (uropatagium). Although previous authors have hypothesised that bat tail membranes perform similar aerodynamic functions to bird tails [9], [10], [11], [12], empirical tests of bat tail aerodynamics have yet to be undertaken. Other studies of bat flight have found marked differences between bat and bird aerodynamics [13], meaning there are potentially significant functional differences between the tails of bats and birds. For example bats seem to generate more complex aerodynamic wakes than birds [14].

**Figure 1:**
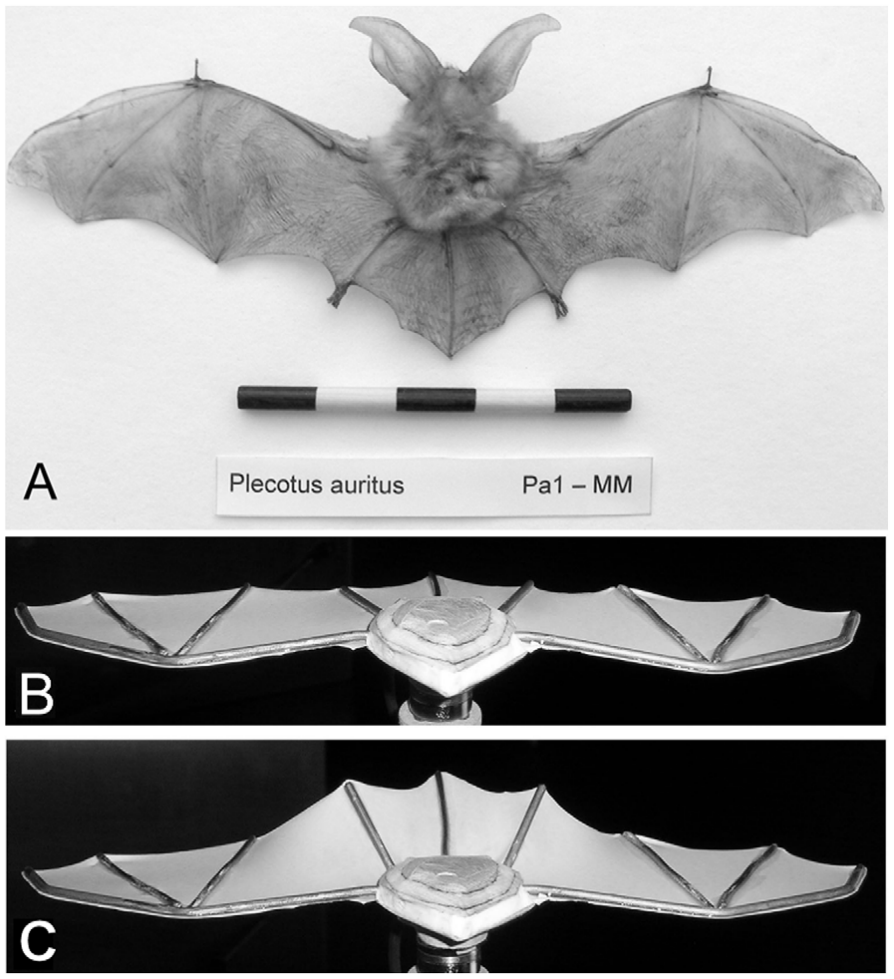
*Plecotus auritus* specimen and the completed wind-tunnel model. A: Dried *P. auritus* bat specimen upon which the bat model was based (Scale bar  =  100 mm). B and C: Completed bat model at the extremes of the leg positions (B: Leg angle (*β*) = 0°, C: Leg angle (*β*) = 60°), showing the effect on the tail membrane angle of attack and the increased wing camber (termed leg-induced wing camber). Leg angle adjustments were made via small screw mountings hidden within the body of the model. The model was mounted upside down in the wind-tunnel to minimise the aerodynamic effect of the wake from the support, since the tail is then deflected away from the support, instead of towards. Note that the large ears of *P. auritus* were excluded from the model, since this investigation was primarily concerned with the aerodynamics of the tail membrane.

Of the 17 families of bats [15] only one family, the old world fruit bats (Pteropodidae), have no real tail membrane. The tail membrane is an extension of the skin between the hind limbs often incorporating the tail vertebrae (Figure 1A). This membrane is usually supported at its rear edge by a thin structure called the calcar, which extends from the ankle joint. The calcar is thought not only to provide structural support for the tail membrane, but also to allow the tail to form a larger aerodynamic surface than if the trailing edge was unsupported [11]. The morphology of the tail is highly variable between species of bats and typically correlates with their foraging style [16]. Insectivorous bats often have long and broad tail membranes that they use as pouches to aid in the capture of insects during flight [17], whereas many nectivorous and frugivorous bats have very reduced tail membranes.

Current understanding of the flight aerodynamics of animal tails is polarised. For example, although delta-wing theory has been used to predict tail performance in birds [8], more recent work [1], [18] suggests this approach is not entirely valid. In bats the tail forms a continuation of the wing membrane (separated by the leg bones) and not a separate lifting surface, therefore, delta wing theory is definitely not applicable. The fact that theoretical approaches based upon aircraft aerodynamics are inadequate when investigating vertebrate tail aerodynamics emphasises the need for new approaches. Accordingly, here we present the first experimental study into the function of bat tails using a wind-tunnel model based on a brown long-eared bat (*Plecotus auritus*). The use of simple physical modelling in biomechanics is a valuable technique as it allows variables to be manipulated in a manner not possible using comparative *in vivo* methods. This approach also allows the performance consequences of each variable to be thoroughly explored [19]. Creating simple models is a well-accepted technique, which has been widely used to gain valuable insights into the flight performance of vertebrates [20], [21], [22], [23]. Furthermore, simple models do not necessarily lead to simple or limited conclusions; for example Taylor et al. [21] used a simple flapping flat plate in a wind tunnel to show that the Strouhal number that all flying and swimming animals cruise at is associated with high power efficiency.

The bat model presented here is necessarily a simplification of a real bat in flight, representing a small gliding bat in steady state aerodynamic conditions. At first glance this may appear at odds with bat biology as most bat species, with the exception of some large bats [24], [25] and the small insectivorous *Pipistrellus pipistrellus* [26], are thought to flap their wings continuously during flight. However, a fixed-wing gliding model can still extend our current understanding of bat flight. Spedding et al. [27] showed that predictions based upon fixed wing data agree well with quantitative observations of flapping flight in birds and that this approach “shows the simplest tenable baseline approximation, upon which more complex and realistic theories might be constructed”. In many ways, therefore, a simple model has advantages over more complicated models by virtue of its simplicity, since this allows any shortcomings in the model to be more easily identified and accounted for. Indeed, the aerodynamic forces and wake produced on an inaccurate flapping model are likely to be more misleading than helpful. We therefore err on the side of simplicity with a gliding bat model that is intended to generate hypotheses for later testing in the field and use our model to provide the first experimental data on the aerodynamic significance of the tail membrane of bats.

## Materials and Methods

### Morphological measurements and model construction

A model for wind tunnel testing was created using detailed morphological measurements taken from a reference specimen held at the Manchester Museum (Manchester, UK) of a brown long-eared bat (*P. auritus,* Figure 1A). *Plecotus auritus* is a slow flying, highly manoeuvrable species which gleans prey from amongst vegetation [28], [29]. All morphological measurements from the museum specimen were taken using digital calipers (16EX 150 mm Prod No: 4102400, Mayr GmbH, Berlin, Germany). The posture of the preserved *P. auritus* specimen, from which the measurements where taken, represents a typical method of stretching out wings of both bird and bats in the field for calculating wing span and area. The model, therefore, provides our best possible representation of the posture of a gliding bat, in the absence of detailed *P. auritus* flight footage, and is consistent with previous work on vertebrate aerodynamics (see Table 1 for model dimensions). The frame of the model was constructed out of plywood, with stiff steel wire to represent the arm, wing, leg and tailbones of the bat. A sheet of 0.1 mm thick latex, cut from a large Semperguard latex glove (Semperit Technische Produkte G.m.b.H, Vienna, Austria), was then stretched over the model frame and glued to the sheet with Cyanoacrylate. Once the glue had dried the model frame was cut out, leaving the stretched latex to form the wings and tail membrane of the bat model (Figure 1B). Latex sheeting was used since this could be tensioned before attachment to the frame, therefore reducing the chance of the trailing edge of the wing fluttering during testing. The latex membrane on the final model was strained approximately 55% in the span-wise direction and 11% in the chord-wise. This corresponds to a pre-stress of approximately 1.0 MPa in the span-wise direction and 0.6 MPa chord-wise, assuming plane stress conditions, a Young's modulus of 1.2 MPa and a Poisson's ratio of 0.5. The actual membrane tension used by these bats during flight is currently unknown, consequently here we made the tension across the membrane as uniform as possible using the materials and methods available to us. One advantage of our modelling technique was that we were able to alter the model as required and in ways not possible with a real bat to ask specific ‘what if’ questions. For example, the tail membrane of the model could be cut out resulting in a morphology that is similar to some nectivorous bats belonging to the Phyllostomidae family, to allow a comparison of the effects of flying with and without a tail membrane. Adjustments to the angle of the tail membrane were made by changing the leg angles via screw fittings hidden within the body of the model. Adjusting the leg positions not only repositioned the tail membrane but also locally changed the camber and angle of attack of the inner surface of the wing (the plagiopatagium) (Figure 1C). Henceforth, we term this effect ‘leg-induced wing camber’. Before wind tunnel testing the corners of the model were rounded and any voids filled with modelling clay to minimise unwanted aerodynamic effects. The large ears of *P. auritus*, which have previously been shown to play a significant role in the aerodynamics of these bats [30] were excluded from the model, as this research focused specifically on the aerodynamics of the tail membrane and therefore it was desirable to avoid interference effects between the ears and tail. Furthermore removing the ears from the model results in a morphology that closely represents a broad range of insectivorous bats, extending the potential relevance of the experimental results.

**Table 1:** Dimensions of wind-tunnel *Plecotus auritus* model, with and without the tail membrane present, showing that removal of the tail membrane reduces the wing area and average wing chord of the model and increases the aspect ratio.

Variable	Model configuration	
	Tail membrane present	Tail membrane removed
Wingspan (m)	0.267	0.267
Wing area (m^2^)	0.0124	0.0108
Aspect ratio	5.7	6.6
Average wing chord (m)	0.0465	0.0404

### Force and moment measurements

**Figure 2:**
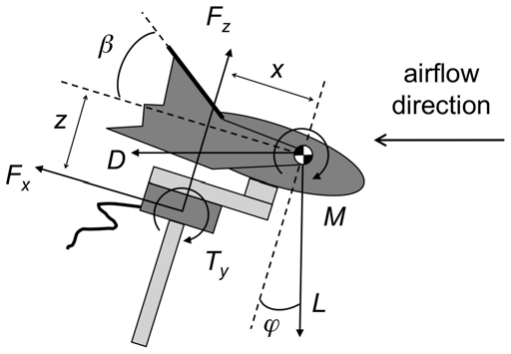
Schematic of the experimental set-up. The relationship between transducer forces and torques (*F*_x_, *F*_z_ and *T*_y_) and lift (*L*), drag (*D*) and pitching moment, due to the body angle (*ϕ*) is illustrated. The leg angle (*β*) and the position of the centre of mass relative to the transducer (*x* and *z*) are also shown. Note that the lift force (*L*) is downwards and the pitching moment (*M*) is clockwise since the model is mounted upside-down.



The force and moment measurements were made using a 6-component force torque transducer (Nano-17, ATI Industrial Automation, USA). Prior to testing the calibration of the transducer was checked using small weights applied in the direction of each axis. Data was acquired using a National Instruments card (Austin, Texas, USA) plugged into a desktop computer. The transducer is manufactured to be accurate down to increments of 0.0125 N (forces) and 0.0625 Nmm (torques). The bat model was mounted onto the transducer via small wooden discs and a thin structural support. This arrangement was then attached to the mast of the wind tunnel at the Université of Liège, Belgium. The wind tunnel working section area of 2×1.5 m is significantly larger than the bat model, removing the potential for unwanted aerodynamic effects induced by the tunnel walls [31]. The bat model was mounted upside-down so that the tail was deflected away from the structural support as opposed to towards it and therefore the effect of the wake from the structural support on the tail membrane aerodynamics was minimised (Figure 1C). The leg angle (*β*) was set relative to the body and the body angle (*ϕ*) was set relative to the oncoming air stream (Figure 2). All angles were set using a large adjustable spirit level, held against the model or support. Data were collected for a model with a tail membrane at leg angles of 0° to 60° in steps of 5° for four separate body angles: −5°, 0°, 5° and 10°. Data were also collected for the same model with the tail membrane removed at 0° and 5° body angles for all leg angles above. The recorded wind speed for all tests fell within the range of 8.6 m/s to 9.3 m/s (Reynolds number range of 2.6×10^4^ to 2.8×10^4^) determined using a pitot tube, which is at the higher end of the natural flight speeds of many insectivorous bats [32]. Although higher than the typical foraging flight speed recorded for *P.auritus* (around 3 m/s) [28], it is comparable to estimates of the commuting speed (6 m/s) in this species [29]. Testing the model at a higher speed has a two-fold benefit. Firstly, the noise/signal ratio received by the force torque transducer is improved, reducing errors and secondly the wind tunnel struggles to produce consistent flow conditions at speeds lower than those tested. Importantly as there is not a significant difference between the Reynolds number of the model testing and that of the natural flight of *P. auritus* the aerodynamic coefficients (i.e. lift coefficient) measured in the wind tunnel will also be applicable to the natural flight of *P. auritus*. Indeed, aerodynamic coefficients are often quoted as being relevant over Reynolds numbers of several orders of magnitude [4]. See Barlow et al. [31] for a complete discussion of the applicability of wind-tunnel test data to real world scenarios, and the importance of maintaining dynamic similarity (i.e. maintaining a constant Reynolds number at low wind speeds). Data were recorded for two hundred samples at a rate of 64 Hz and averaged to give a steady-state reading. The lift and drag readings were corrected at each body angle, to ensure that they were relative to the incoming wind direction using the following equations:

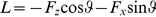
(1)

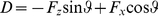
(2)


Where *L* is the lift, *D* the drag, *F_x_* and *F_z_* the transducer forces and *ϕ* the body angle (Figure 2). The pitching moment (defined as nose up positive) was relocated from the force torque transducer to the centre of mass of the bat model using



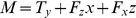
(3)


Where *M* is pitching moment, *T_y_* the transducer torque, *x* and *z* the location of the centre of mass of the bat relative to the transducer (Figure 2). The location of the centre of mass of the bat model relative to the force balance was calculated by firstly weighing the model. Then the model was attached to the transducer and force and torque measurements taken at several different body angles whilst the tunnel was turned off. These measurements were then used to set up simultaneous equations, which were solved to find the centre of mass of the model, relative to the transducer (i.e. *x* and *z*). The location of the centre of mass on the bat model corresponded well with methods used to estimate the centre of mass of live bats [12]. The lift, drag and pitching moment were converted into non-dimensional aerodynamic coefficients using the following equations:



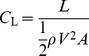
(4)

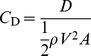
(5)

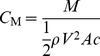
(6)


Where *C*_L_, *C*_D_ and *C*_M_ are the lift, drag and pitching moment coefficients respectively, *ρ* is the air density, *V* the air speed, *A* the wing area and *c* the average wing chord. Finally the lift to drag (*L/D*) ratio of the model was calculated for each test position since this ratio gives a good indication of overall aerodynamic performance.

### Statistical analysis

ANCOVA was used to determine whether the different model configurations (body angles of 0 and 5°, and with, and without a tail membrane present) changed the relationship between aerodynamic parameters and leg angle. Tukey's post hoc tests were used to indentify specific differences between the four model configurations used. All statistical tests were preformed using the statistics toolbox for MATLAB® R2009a (MathWorks, Natick, Massachusetts, USA).

## Results

During wind tunnel testing little aero-elastic deformation of the model's latex wing membranes or wing struts was observed. There was also no obvious fluttering of the trailing edge of the membrane. The only deformation of the latex membrane observed was the local increase in wing camber (leg-induced wing camber) due to the repositioning of the legs (Figure 1C), previously discussed in the methods.

**Figure 3:**
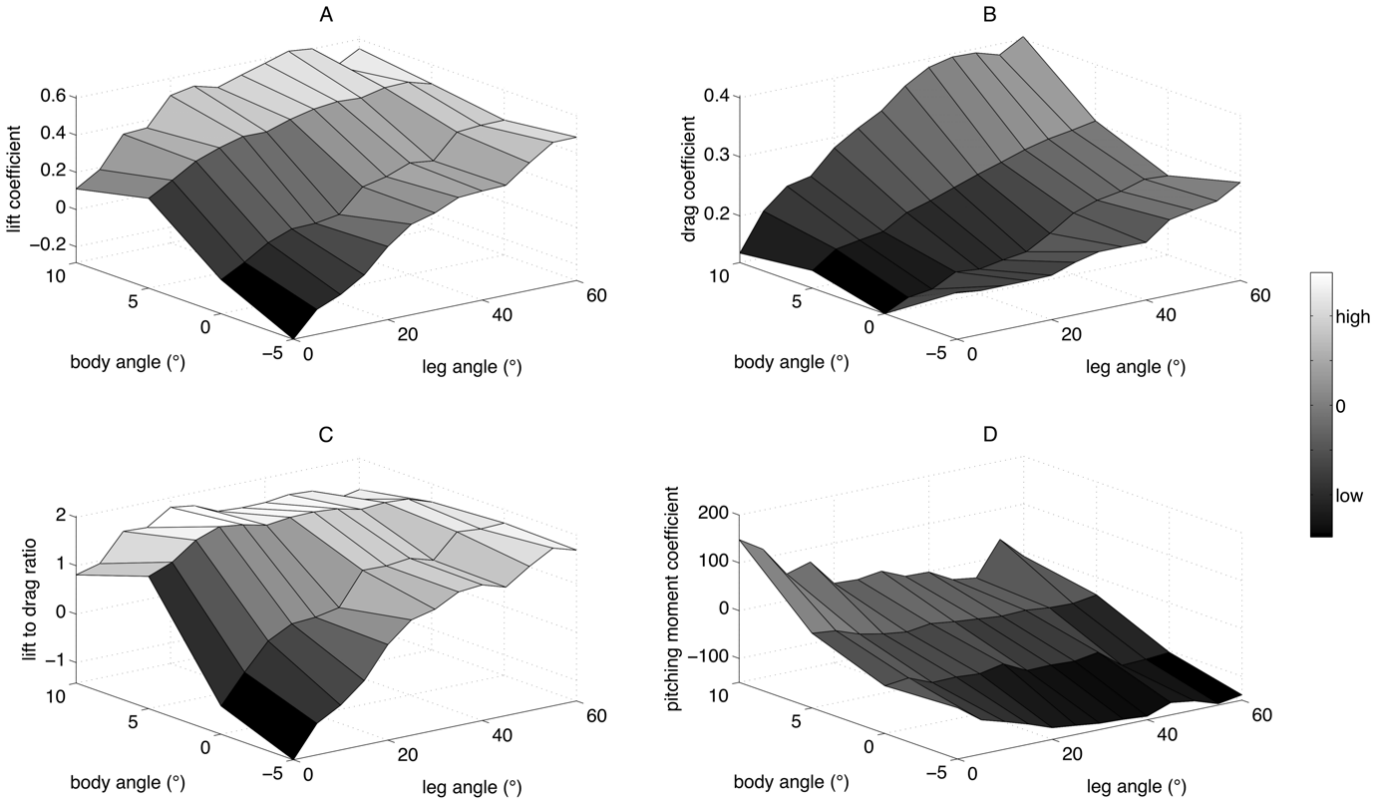
Aerodynamic force and moment coefficients produced by the bat wind-tunnel model. Effect of both leg angle and body angle on the lift coefficient (A), drag coefficient (B), lift to drag ratio (C) and the pitching moment coefficient (D) generated by the bat model with tail membrane present during wind-tunnel tests. Darker grey indicates lower values, while lighter grey higher values.

**Figure 4:**
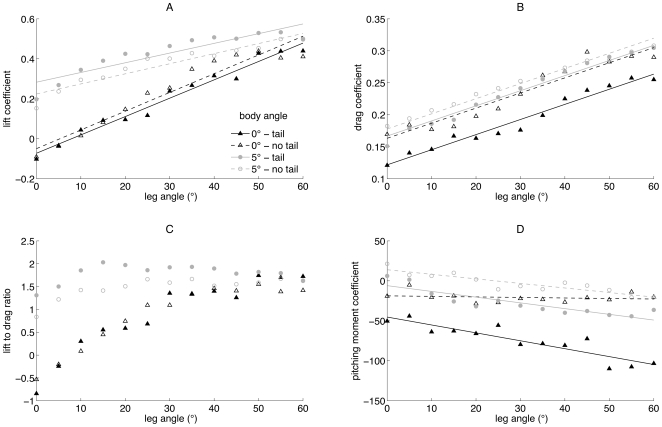
Comparison of the bat model's aerodynamic coefficients with and without a tail membrane. Lift coefficient (A), drag coefficient (B), lift to drag ratio (C) and pitching moment coefficient (D) produced by bat model for two body angles (0° and 5°) at all leg angles, with and without a tail membrane. Solid lines represent model with tail membrane. Dashed lines represent model without tail membrane. Black lines are for body angle of 0° and grey lines for 5°.

The *C*_L_ and *C*_D_ produced by the bat model with a tail membrane follow similar general trends with body angle and leg angle (Figure 3A and B). As leg and body angle increased *C*_L_ and *C*_D_ also increased. An ANCOVA (Figure 4A) confirmed that *C*_L_ increased with both leg angle (*β*) and also changed with bat model configuration (body angle and presence/absence of the tail membrane), and the incremental change (i.e. the slope) in *C*_L_ with leg angle differed between the model configurations (leg angle: *F*_1,44_ = 521.53, *p*<0.001; configuration: *F*_3,44_ = 88.18, *p*<0.001; configuration*leg angle: *F*_3,44_ = 16.11, *p*<0.001). Furthermore, it is clear from Figure 4A and was confirmed by Tukey's post-hoc test, that the relationship between *C*_L_ and leg angle was similar for the model with and without a tail membrane; only body angle had an effect. The *C*_L_ produced by the bat model is, therefore, increased by both leg angle and body angle, but the removal of the tail membrane has no impact.

The *C*_D_ was always positive and tended to increase with body and leg angle (Figure 3B). ANCOVA showed that there was no difference in the slopes of *C*_D_ against leg-angle for any of the model configurations (configuration*leg angle: *F*_3,44_ = 2.29, *p* = 0.0916). Accordingly, simplifying the ANCOVA to assume parallel lines (i.e., no difference in slopes) showed that *C*_D_ increased with leg angle and changed with model configuration (leg angle: *F*_1,47_ = 1130.68, *p*<0.001; configuration: *F*_3,47_ = 87.36, *p*<0.001). Figure 4B shows, and Tukey's post hoc test confirmed, that contrary to the limited effect on the *C*_L_, *C*_D_ is increased by the removal of the tail membrane from bat model. The *C*_D_, therefore, increases with both increasing leg angle and body angle, and further increases with the removal of the tail membrane from the model.

The *L/D* ratio (Figure 3C and 4C) has a more complex relationship with body and leg angle. These data were not analysed with an ANCOVA since the *L/D* ratio is derived from both the *C*_L_ and *C*_D_, which are have already been analysed. It is apparent, however, that the highest *L/D* ratio was produced at approximately a body angle of 5° and a leg angle of 20°.

Decreasing body angle and increasing leg angle caused the pitching moment coefficient to decrease (Figure 3D). This does not mean, however, that the pitching moment coefficient tended to zero, in fact it became negative (i.e. a higher nose-down pitching moment) at the lowest body and highest leg angles. An ANCOVA showed that the pitching moment coefficient differed between model configurations and increased with leg angle, and the incremental change in pitch moment coefficient with leg angle also differed between model configurations (configuration: *F*_3,44_ = 218.75, p<0.001; leg angle: *F*_1,44_ = 111.93, *p*<0.001; configuration*leg angle: *F*_3,44_ = 12.23, p<0.001). Removal of the tail membrane from the model has a profound affect on the pitching moment produced. (Figure 4D). Tukey's post hoc test of the original ANCOVA showed that removing the tail membrane substantially reduced the level of nose-down pitching produced by the bat model.

## Discussion

Changes in leg angle had a significant impact on the aerodynamic performance of the bat model (Figure 3). These leg angle induced aerodynamic effects are likely to be due to two main factors; increased leg-induced wing camber (i.e. locally increased camber and angle of attack of the inner wing surface of the model) and an increase in the angle of attack of the tail membrane. Both have a different impact on the bat model's aerodynamics and therefore different implications for *P. auritus* flight performance.

One of the most critical issues of flight performance is the trade-off between stability and manoeuvrability [33]. The pitching moment coefficient results (Figure 3D) are important in defining the model's stability. First, for almost all leg angles the pitching moment coefficient around the centre of gravity increases with body angle. This means that the bat model is statically unstable. Consider the case where a bat is in equilibrium, i.e. the pitching moment is zero (*M* = 0) and lift equals weight (*L-W* = 0). Then in general, a statically stable bat would be defined by



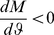
(7)


(i.e. the slope of the equation describing the relationship between the pitching moment coefficient and body angle should be negative). In this statically stable case, an increase in body angle due to, for example atmospheric turbulence will be corrected by an accompanying decrease in pitching moment and the bat will return to equilibrium position. However, the results of the pitching moment (Figure 3D) for the bat model clearly demonstrate that



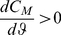
(8)


i.e. the slope of the relationship between pitching moment coefficient (and therefore the pitching moment) and body angle is positive. In this case any increase in body angle will tend to be exacerbated by the increase in pitching moment, which will in turn increase body angle further making the bat model statically unstable. Second, the pitching moment results show that the slope of the surface is not significantly affected by the leg angle (Figure 3D). In other words, leg angle doesn't change the degree of instability of the bat model. The main effect of leg angle is to decrease pitching moment at all body angles. This is consistent with aerodynamic theory, which states that increasing wing camber causes increasing nose-down pitching moment, i.e. a negative nose-up moment [34]. Interestingly the inclusion of a tail membrane on the model exacerbates the increases with body angle of the pitching moment produced by the model (Figure 4D). Therefore, equation 8 would predict the model with a tail membrane is more unstable than the model without a tail membrane. In many ways this is counterintuitive since an aerodynamically active surface behind the centre of mass, generally aids stability. The tail membrane of bats, however, is not a separate aerodynamic surface but rather an extension of the wing membrane separated only by the leg bones, and therefore cannot be considered as a separate aerodynamic surface.

The most obvious explanation for the static instability of the bat model is that the centre of pressure of the wing (the point where the aerodynamic forces act) lies in front of the centre of mass. Of course, real bats can flap and deform their wings in a complex manner [35] and small modifications of the sweep angle of the wing could shift the position of the centre of pressure behind the centre of mass and thus produce a statically stable configuration [24], [36]. Nevertheless, the centre of mass of the model is consistent with estimates for real bats [12] and suggests a gliding *P. auritus* configuration is statically unstable. A lack of static stability is not necessarily undesirable. Acrobatics aircraft are often neutrally stable (on the border between static stability and instability) as this increases their flight agility and the ability of the pilot to perform stunts [37].

Repositioning the tail membrane by increased leg angle, increases the pitching moment coefficient produced by the model, compared to the model without a tail membrane (Figure 4D). Therefore, the tail membrane could be an important structure for improving manoeuvrability and agility of *P. auritus*, particularly around the pitch axis. The wings of bats are well positioned to produce the necessary rolling and yawing moments around the centre of mass required for many manoeuvres [38]. However, wings are poorly positioned to produce large pitching moments around the centre of mass, since the quarter lifting line of a wing (i.e. the line which the lift force acts through) lies close to the pitching axis, which passes through the centre of mass [5]. This is a desirable scenario for most steady state horizontal flight, when average pitching moment over several flaps will tend to zero. However, during manoeuvres, a large pitching moment may be desirable so that the lift and thrust force can quickly be redirected and a turn made. Indeed, studies of manoeuvring bats have shown that the manoeuvres involve complex kinematics and changes around more than just the roll axis [38], [39]. Roll acceleration is clearly important for initiating and completing manoeuvres and several taxa that forage close to vegetation (for example *Eptesicus nilssoni* and *Pipistrellus pipistrellus*) have specialisations in wing morphology, such as broad wing tips, to enhance the aerodynamic rolling moment generated by their wings [40]. However, during the banked phase of a turn the control of both the yawing and pitching moment (in addition to the rolling moment) will be essential if the manoeuvre is to be completed successfully. Therefore, possibly one of the important functions of a bat tail membrane (and indeed bird tails) is to control the pitching moment produced around the centre of mass, allowing control of the orientation of the lift forces and therefore more precise manoeuvres.

Removing the tail membrane from the bat model has no significant impact on the *C*_L_ produced by the model (Figure 4A). This suggests for the aerodynamic features tested on the bat model that the leg-induced wing camber is a more important feature than the angle of attack of the tail membrane for controlling the level of lift produced. This doesn't mean the tail membrane has no role in affecting lift production; rather that the leg-induced wing camber seems to have a more significant effect. This is a slightly surprising result since bird tails clearly do have an important lifting function, particularly at lower speeds [1], [2], and a similar role had been hypothesised for the bat tail membrane [9], [10]. However, a bird can easily change the area of its tail by fanning out feathers, therefore changing its aerodynamic function to suit the current flight speed. For example, when birds come in to land they fan out their tail and increase its angle of attack, whereas during faster flight the tail is generally more furled [9]. For bats changing the area of their tail membrane to suit different flight speeds is not such a simple task; perhaps they can achieve some level of tail area control by appropriate positioning of their hind legs, however this remains to be tested. Furthermore, since leg position will influence both the positioning of tail membrane and the amount of leg-induced wing camber, it is impossible for the bat to manipulate the aerodynamics of one without affecting the other. In this sense the name tail membrane is perhaps a misnomer, since although the membrane encompasses the tail vertebrae, it is more akin to a wing flap.

The presence of a tail membrane on the model bat was shown to reduce the *C*_D_ produced (Figure 4B). Suggesting that tail membrane may act as a splitter plate, streamlining the body of the bat, as has been suggested previously for bird tails [3]. Furthermore, this potential drag reduction role may help to explain why many bat species that lack a large tail membrane, still posses small fringes of skin around the back of the body and legs.

Increasing leg-induced wing camber via appropriate leg positioning impacts the lift and drag coefficients produced by the bat model (Figure 3). The control of wing camber in flying bats is clearly important for controlling the magnitude of the lift and drag produced and is known to vary in a complex manner across the wing surface during each stroke [41]. Furthermore, camber has long been recognised in the aircraft aerodynamic literature as a key parameter in the aerodynamic performance of aircraft wings. Therefore, it is not surprising that the ability of bats to camber their wing surface is also recognised to have a distinct impact on their flight performance and foraging behaviour [32], [42]. Indeed, it is not only the control of wing camber, but the deformation of the flexible membrane in response to aerodynamic loads, that has been shown to affect a bat's aerodynamic performance [22], [43]. This automatic cambering behaviour of the wing skin is thought to delay the onset of stall [44]. Very little aero-elastic deformation of the latex membrane, however, was observed on the wind tunnel model tested here. The level of camber present on a bat's wing has a critical impact on its aerodynamic performance and our model results show that bats may partly control their wing camber through appropriate positioning of the legs.

Compared to experimental results of the gliding flight of live birds and bats in wind tunnels, the model's gliding performance is poor. The lift to drag ratio of the model doesn't get higher than around 2 (Figure 3C), whereas the dog-faced bat *Rousettus aegyptiacus* reached a maximum of 6.8 during glide tests in a tilting wind-tunnel [24]. This is not surprising since the bat model is necessarily a simplification of live bats and only the function of the tail membrane (and not the wings) was being investigated. Furthermore, the model was designed to enable testable hypotheses to be generated rather than provide quantitative aerodynamic performance parameters for a gliding bat planform.

Given that tails appear to improve flight performance of *P. auritus* it is interesting that many species of fruit bats lack a tail membrane. Fruit bats, however, are unlikely to require a high level of flight performance since the vast majority of their foraging time is spent either climbing in the trees, or in direct flight between roosts and foraging areas. The additional control of pitching moment and hence flight performance that the model tests indicate repositioning a tail membrane produces (Figure 4) may not therefore be required for foraging fruit bats. Therefore, other ecological pressures such as roosting behaviour [11] may dictate the presence or absence of the tail membrane. Aerial insectivores, on the other hand, require high levels of flight performance since they catch prey on the wing or amongst the clutter of vegetation. For the gleaning and slow flying hawking bats, manoeuvrability (i.e. the ability to perform tight turns) is a key factor that will influence foraging success. Manoeuvrability is likely to be best in bats possessing the lowest wing loading and an ability to sustain high *C*_L_ [45]. Therefore having a large tail membrane is likely to confer several key flight benefits. For example, the increase in wing area provided by having a tail membrane will reduce wing loading and therefore potentially improve manoeuvrability. A large tail membrane will also potentially offer a foraging advantage for bats that use the tail membrane as an insect capturing pouch [17], presenting a large area with which to snare prey. The data here highlights a potential role for the tail membrane in flight control, however whether this role is the primary function of the tail or a secondary function to improving prey capture is difficult to clarify. High speed footage of bats using their tail in flight and for prey capture may help distinguish between these functions. Altering the positioning of the hind legs potentially allows additional control of the wing camber for all bat species and therefore afford bats a level of either passive or active control of the lift and drag forces. Birds, on the other hand, are limited in their ability to adjust wing camber since feathers are relatively stiff structures and are not connected to the hind legs or tail.

Our model data here presents the first experimental evidence for a flight function of the bat tail membranes and provides a foundation for future research efforts. It would, for example, be very interesting to study whether bats actively control their leg position during flight as the model results suggests since potentially this is similar to a bird's control of tail position and furl which allows them to actively influence their aerodynamic performance. The alternative to active control is passive positioning of the legs and tail membrane driven by the inherent aerodynamic and inertial loads from the wings and body of the bat. From the results of the *P. auritus* model we conclude that the tail membrane of many bats (since many have wings and tails morphologically similar to *P. auritus*) has a flight control function and hypothesise that:

1. bats will actively control leg position (and hence tail position and leg-induced wing camber), since this will allow greater control over their flight and consequently, their foraging performance.
2. bats will rapidly reposition their legs and tail, coincident with aerial manoeuvres.
3. bats with the longest legs and largest tail membranes will be the most manoeuvrable.

## References

[1] Evans MR (2003). Birds' tails do act like delta wings but delta-wing theory does not always predict the forces they generate.. Proceedings of the Royal Society of London B Biological Sciences.

[2] Maybury WJ, Rayner JMV, Couldrick LB (2001). Lift generation by the avian tail.. Proceedings of the Royal Society of London B Biological Sciences.

[3] Maybury WJ, Rayner JMV (2001). The avian tail reduces body parasite drag by controlling flow separation and vortex shedding.. Proceedings of the Royal Society of London B Biological Sciences.

[4] Vogel S (1994). Life in Moving Fluids..

[5] Thomas ALR, Taylor GK (2001). Animal Flight Dynamics I. Stability in Gliding Flight.. Journal of Theoretical Biology.

[6] Sachs G (2007). Tail effects on yaw stability in birds.. Journal of Theoretical Biology.

[7] Sachs G (2007). Why Birds and Miniscale Airplanes Need No Vertical Tail.. Journal of Aircraft 44:.

[8] Thomas ALR (1993). On the Aerodynamics of Birds' Tails.. Philosophical Transactions of the Royal Society of London B Biological Sciences B.

[9] Norberg UM (1990). Vertebrate Flight..

[10] Lawlor TE (1973). Aerodynamic Characteristics of Some Neotropical Bats.. Journal of Mammalogy.

[11] Vaughan TA, Wimsatt WA (1970). Flight patterns and aerodynamics.. Biology of bats.

[12] Bullen R, McKenzie NL (2001). Bat airframe design: flight performance, stability and control in relation to foraging ecology.. Australian Journal of Zoology.

[13] Hedenström A, Johansson LC, Spedding GR (2009). Bird or bat: comparing airframe design and flight performance.. Bioinspiration & Biomimetics.

[14] Hedenström A, Johansson M, Wolf M, von Busse R, Winter Y (2007). Bat Flight Generates Complex Aerodynamic Tracks.. Science.

[15] Nowak RM (1994). Walker's Bats of the World..

[16] Gardiner JD, Codd JR, Nudds RL (2011). An association between ear and tail morphologies of bats and their foraging style.. Canadian Journal of Zoology.

[17] Webster FA, Griffin DR (1962). The role of the flight membrane in insect capture by bats.. Animal Behaviour.

[18] Evans MR, Rosén M, Park KJ, Hedenström A (2002). How do birds' tails work? Delta-wing theory fails to predict tail shape during flight.. Proceedings of the Royal Society of London B Biological Sciences.

[19] Koehl MAR (2003). Physical Modelling in Biomechanics.. Philosophical Transactions of the Royal Society of London B Biological Sciences.

[20] Wilkinson MT, Unwin DM, Ellington CP (2006). High lift function of the pteroid bone and forewing of pterosaurs.. Proceedings of the Royal Society of London B Biological Sciences.

[21] Taylor GK, Nudds RL, Thomas ALR (2003). Flying and swimming animals cruise at a Strouhal number tuned for high power efficiency.. Nature.

[22] Galvao R, Israeli E, Song A, Tian X, Bishop K (2006). The Aerodynamics of Compliant Membrane Wings Modeled on Mammalian Flight Mechanics.. AIAA paper.

[23] Song A, Breuer K (2007). Dynamics of a Compliant Membrane as Related to Mammalian Flight.. AIAA paper.

[24] Pennycuick CJ (1971). Gliding Flight of the Dog-Faced Bat *Rousettus Aegyptiacus* Observed in a Wind Tunnel.. Journal of Experimental Biology.

[25] Norberg UM, Brooke AP, Trewhella WJ (2000). Soaring and non-soaring bats of the family pteropodidae (flying foxes, Pteropus spp.): wing morphology and flight performance.. Journal of Experimental Biology.

[26] Thomas ALR, Jones G, Rayner JMV, Hughes PM (1990). Intermittent gliding flight in the pipistrelle bat (*Pipistrellus pipistrellus*)(Chiroptera:Vespertilionidae).. Journal of Experimental Biology.

[27] Spedding GR, Hedenstrom AH, McArthur J, Rosen M (2008). The implications of low-speed fixed-wing aerofoil measurements on the analysis and performance of flapping bird wings.. Journal of Experimental Biology.

[28] Swift S (1998). Long-eared bats..

[29] Howard RW (1995). Auritus - A natural history of the brown long-eared bat..

[30] Gardiner J, Dimitriadis G, Sellers W, Codd J (2008). The aerodynamics of big ears in the brown long-eared bat *Plecotus auritus*.. Acta Chiropterologica.

[31] Barlow JB, Rae WH, Pope A (1999). Low-speed wind tunnel testing..

[32] Baagøe HJ, Fenton MB, Racey PA, Rayner JMV (1987). The Scandinavian bat fauna.. Recent advances in the study of bats.

[33] Maynard Smith J (1952). The Importance of the Nervous System in the Evolution of Animal Flight.. Evolution.

[34] Anderson JD (2007). Fundamentals of Aerodynamics..

[35] Swartz S, Iriarte-Diaz J, Riskin D, Song A, Tian X (2007). Wing Structure and the Aerodynamic Basis of Flight in Bats.. AIAA paper.

[36] Weyl AR (1945). Stability of tailless aeroplanes.. Aircraft Engineering.

[37] Stinton D (2001). The Design of the Aeroplane..

[38] Norberg UM (1976). Some advanced flight manoeuvres of bats.. Journal of Experimental Biology.

[39] Iriarte-Diaz J, Swartz SM (2008). Kinematics of slow turn maneuvering in the fruit bat *Cynopterus brachyotis*.. Journal of Experimental Biology.

[40] Thollesson M, Norberg UM (1991). Moments of Inertia of Bat Wings and Body.. Journal of Experimental Biology.

[41] Swartz S, Bishop K, Aguirre M, Zubaid A, McCracken GF, Kunz TH (2006). Dynamic Complexity of Wing Form in Bats: Implications for Flight Performance.. Functional and Evolutionary Ecology of Bats.

[42] Stockwell EF (2001). Morphology and flight manoeuvrability in New World leaf-nosed bats (Chiroptera: Phyllostomidae).. Journal of Zoology (London).

[43] Song A, Tian X, Israeli E, Galvao R, Bishop K (2008). Aeromechanics of Membrane Wings with Implications for Animal Flight.. AIAA Journal.

[44] Shyy W, Lian Y, Tang J, Viieru D, Liu H (2008). Aerodynamics of low Reynolds number flyers..

[45] Norberg UM, Rayner JMV (1987). Ecological morphology and flight in bats (Mammalia; Chiroptera): Wing adaptations, flight performance, foraging strategy and echolocation.. Philosophical Transactions of the Royal Society of London B Biological Sciences B.

